# Supplementary material for: Anti-Apolipoprotein A-1 IgG Influences Neutrophil Extracellular Trap Content at Distinct Regions of Human Carotid Plaques
Source: Int J Mol Sci. 2020 Oct 19;21(20):7721. doi: 10.3390/ijms21207721 (PMC7588926; doi:10.3390/ijms21207721)
Supplement: Supplementary file 1 [file ijms-21-07721-s001.pdf]

Figure S1

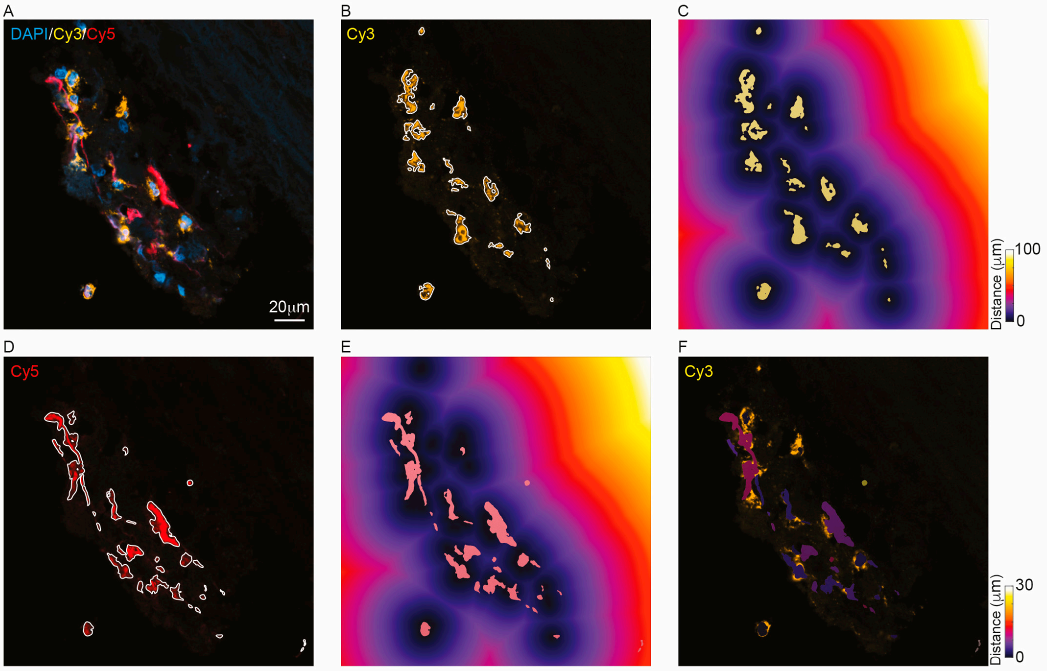

Fig. S1. Estimation of the h3cit maximum distance to neutrophils.
(A) Overlay of the DAPI (blue), neutrophils labeled with Cy3 (yellow) and H3cit labeled with (Cy5). **(B)** Neutrophils boundary is indicated in white. **(C)** Distance map to the neutrophils (yellow surfaces) expressed in µm is color coded from dark purple (the closest) to white (the furthest). **(D)** H3cit presence is indicated in white. **(E)** Relative position of the H3cit with respect to the distance from the neutrophils (same map than in (C)). **(E)** Color-coding of each individual H3cit region based on the neutrophil maximum distance within this same region. The H3cit-neutrophil distance expressed in µm is color-coded from dark purple (the closest) to white (the furthest).

Supplemental Figure 1. Estimation of H3Cit maximum distance to neutrophils. (**A**) Overlay of DAPI (blue), neutrophils labelled with Cy3 (yellow) and H3Cit labelled with Cy5 (red). Neutrophils boundary is indicated in white. (**C**) Distance map to the neutrophils (yellow surfaces) expressed in µm is coded from dark purple (the closest) to white (the furthest). (**D**) H3Cit presence is indicated in white. (**E**) Relative position of the H3Cit with respect to the distance from the neutrophils (same map than in (C)). (**F**) Color-coding of each individual H3Cit region based on neutrophil maximum distance within this same region. The H3Cit-neutrophil distance expressed in µm is color-coded from dark purple (the closest) to while (the furthest).
